# Ameliorating Adriamycin-Induced Chronic Kidney Disease in Rats by Orally Administrated Cardiotoxin from *Naja naja atra* Venom

**DOI:** 10.1155/2014/621756

**Published:** 2014-04-30

**Authors:** Zhi-Hui Ding, Li-Min Xu, Shu-Zhi Wang, Jian-Qun Kou, Yin-Li Xu, Cao-Xin Chen, Hong-Pei Yu, Zheng-Hong Qin, Yan Xie

**Affiliations:** ^1^The First Affiliated Hospital of Soochow University, Suzhou, Jiangsu 215006, China; ^2^Department of Pharmacology and Laboratory of Aging and Nervous Diseases, Soochow University School of Medicine, Suzhou 215123, Jiangsu, China; ^3^The Second Affiliated Hospital of Soochow University, Suzhou, Jiangsu 215004, China

## Abstract

Previous studies reported the oral administration of *Naja naja atra* venom (NNAV) reduced adriamycin-induced chronic kidney damage. This study investigated the effects of intragastric administrated cardiotoxin from *Naja naja atra* venom on chronic kidney disease in rats. Wistar rats were injected with adriamycin (ADR; 6 mg/kg body weight) via the tail vein to induce chronic kidney disease. The cardiotoxin was administrated daily by intragastric injection at doses of 45, 90, and 180 **μ**g/kg body weight until the end of the protocol. The rats were placed in metabolic cages for 24 hours to collect urine, for determination of proteinuria, once a week. After 6 weeks, the rats were sacrificed to determine serum profiles relevant to chronic kidney disease, including albumin, total cholesterol, phosphorus, blood urea nitrogen, and serum creatinine. Kidney histology was examined with hematoxylin and eosin, periodic acid-Schiff, and Masson's trichrome staining. The levels of kidney podocin were analyzed by Western blot analysis and immunofluorescence. We found that cardiotoxin reduced proteinuria and can improve biological parameters in the adriamycin-induced kidney disease model. Cardiotoxin also reduced adriamycin-induced kidney pathology, suggesting that cardiotoxin is an active component of NNAV for ameliorating adriamycin-induced kidney damage and may have a potential therapeutic value on chronic kidney disease.

## 1. Introduction

Chronic kidney disease is characterized by reduced glomerular filtration and persistent massive proteinuria. Because of its increasing morbidity and mortality, chronic kidney disease has become an important research field [1]. Adriamycin induced nephropathy is considered to be a classic rat model of chronic kidney disease [2]. Glomerular filtration barrier damage plays a critical role in proteinuria in chronic kidney disease [3], which contains fenestrated endothelium, glomerular basement membrane (GBM), and slit diaphragm (SD) [4]. In particular, SD is the most important component of the glomerular filtration barrier. Previous studies have shown that podocin encoded by NPHS2 [5] is a critical component protein in slit diaphragm [6, 7], and that reduced expression of podocin correlates to the severity of proteinuria [8].

Cobra venom is a complex of many proteins, peptides, and enzymes that contains cardiotoxin, neurotoxin, PLA2, cytotoxin, nerve growth factor, lectins, and disintegrins and exhibits a variety of pharmacological and toxic activities including anti-inflammatory, bactericidal, analgesic, anticancer, platelet-aggregation inhibition, and anticoagulant, myotoxic, neurotoxic, hypotension, and haemolytic actions [9, 10]. Cardiotoxin accounts for approximately 25–50% of the components of* Naja naja atra* venom (NNAV) [11]. Previous studies have already demonstrated that cardiotoxin contains six isoforms: CTX I, CTX II, CTX III, CTX IV, CTX V [12, 13], and CTX*n* [14]. Various Studies have already proven that cardiotoxins have analgesic [15], anti-inflammatory, antiapoptotic, anticancer, and bactericidal actions [16, 17]. Our previous studies have demonstrated that NNAV has a protective effect on adriamycin nephropathy [18] and diabetic nephropathy [19]. Our recent studies suggest that neurotoxin from NNAV protects kidney against adriamycin-induced neuropathy (unpublished data). We speculated that cardiotoxin might also mediate the protective effects of NNAV on chronic kidney disease.

## 2. Materials and Methods

### 2.1. Animals

All studies were performed in accordance with the National Institutes of Health Guide for the Care and Use of Laboratory Animals (National Research Council, 1996) and were approved by the Soochow University Animal Care and Use Committee. Male Wistar rats weighing 140–160 grams were obtained from the Shanghai SLAC Laboratory Animal Co., Ltd. (number: 2007000546473). All rats were kept in a climate controlled room (12 hour light/dark cycle, temperature 22–25°C, humidity 50–60%) with adequate standard laboratory food and tap water. During the experiment, body weight was measured once a week.

### 2.2. Degradation Assay of Cardiotoxin in Stomach and Artificial Gastric Juice

After deprivation of food and water for 24 h, the ICR male mice (number: 2007000564768, SLAC, Shanghai) were ligated pylorus under anesthesia. Then the mice were administrated cardiotoxin at dose of 20 mg/kg body weight for 15 minutes. The gastric juice (GJ) was extracted out and the supernatant was separated for assay of cardiotoxin after centrifugation at 12,000 rpm for 10 minutes at 4°C. For in vitro assay, cardiotoxin was incubated in artificial gastric juice (AGJ) containing 1% pepsin (0685, AMRESCO, LLC) and 1.64% dilute hydrochloric acid (pH 1–1.2) or in distilled water with pH adjusted to 1.2 with HCl for 15 minutes. After centrifugation at 12,000 rpm for 10 minutes at 4°C, the supernatant was separated for degradation assay. The supernatants were loaded into PAGEL and separated by electrophoresis and dyed with imperial protein stain (24615, Thermo Scientific).

### 2.3. Drug Administration

Adriamycin (abbreviation: ADR; also called doxorubicin hydrochloride) was obtained from Shenzhen Main Luck Pharmaceuticals Inc. (Shenzhen, China) and cardiotoxin (abbreviation: CTX), purchased from Orientoxin Biotechnology Co., Ltd. (Laiyang, Shandong Province, China). The purity of CTX was 95.8% (number: 120301) in which the main isomer was CTX IV (99.6%, confirmation number: 737178981). After a few days of feeding adaptation, chronic kidney disease was induced in rats by a single intravenous injection of ADR (6 mg/kg body weight [20], dissolved in sterile 0.9% saline solution). The rats were then randomly divided into four groups: one adriamycin nephropathy group (model group) and the other three treatment groups received intragastrically administrated cardiotoxin at a dose of 45 *μ*g/kg, 90 *μ*g/kg, and 180 *μ*g/kg. The doses of cardiotoxin were decided based on previous studies on rheumatoid arthritis (RA) and the initial preexperiment on adriamycin nephropathy. The normal control group was injected with sterile saline via the tail vein and with intragastrically administered distilled water.

### 2.4. Urine Collection

Throughout the experiment, all rats were placed in metabolic cages with adequate standard laboratory food and tap water, to collect 24-hour urine on a weekly basis. The 24-hour urine total protein concentration of collected urine was then analyzed with the Coomassie brilliant blue protein assay kit [21] (Nanjing Jiancheng Bioengineering Institute, China) and measured with an ultraviolet spectrophotometer (UV-2600, Shimadzu, Tokyo, Japan).

### 2.5. Blood Serum Indexes Measurement

After intragastric administration for six weeks, all rats were anesthetized by intraperitoneal injection of pentobarbital and sacrificed after abdominal aortic blood collection. Serum was separated by centrifugation at 3,000 rpm for 15 minutes at 4°C and stored at −80 degrees for backup. Serum biological indexes of albumin (ALB), globulin (GLB), albumin/globulin ratio (ALB/GLB), triglyceride (TG), total cholesterol (TC), phosphorus (P), sodium (Na), blood urea nitrogen (BUN), and serum creatinine (SCr) were detected by an automatic biochemistry analyzer (Mindray BS-800, Shenzhen, China).

### 2.6. Light Microscopy and Immunofluorescence

The kidneys were removed, weighed, and fixed in 10% formalin to make paraffin-embedded tissue and then cut into 3 *μ*m thickness slices. These sections were stained with hematoxylin and eosin (HE), periodic acid-Schiff (PAS), and Masson's trichrome and then observed by Olympus light microscopy (Olympus, Tokyo, Japan) with a high-resolution digital camera system. Paraffin slices were immersed in xylenes, in decreasing grades of ethanol (100% to 75%) and deionized water for deparaffinizing and rehydrating, and boiled in citrate buffer for antigen retrieval for 15 minutes. Then the slices were blocked in PBS, containing 5% horse serum albumin and 0.4% Triton X-100 for 2.5 hours at room temperature. Then the slices were incubated by primary antibody (rabbit polyclonal antipodocin antibody, sc-21009, SANTA CRUZ BIOTECHNOLOGY, USA) overnight at 4°C. After washing slices by PBS which contains 0.2% Triton X-100 for 10 minutes × 3 times, the slices were incubated with Cy3-conjugated affinipure donkey anti-rabbit IgG (1 : 1000; Jackson ImmunoResearch Laboratories, West Grove, PA, USA) for 1 hour at room temperature. After incubating with 4,6-diamidino-2-phenylindole (DAPI) for 10 minutes, the slices were dehydrated in increasing ethanol grades and cover-slipped with fluoromount Aqueous Mounting Medium (Sigma, F4680). The slices were analyzed by a laser-scanning confocal unit (Zeiss LSM 710, Carl Zeiss, Jena, Germany).

### 2.7. Western Blot Analysis

The renal cortices were ground with the lysate (Tris pH 7.4, deoxysodium cholate, Triton X-100, NaCl, 1% SDS, EDTA-2Na) containing protease inhibitors (Protease Inhibitor Cocktail Tablets, Roche, Mannheim, Germany). After centrifugation at 15,000 rpm for 15 minutes at 4°C, the supernatant was separated for qualitative detection of protein. The total protein concentration was determined by using a BCA kit (Pierce Biotechnology, Waltham, MA, USA). The same amount of protein was separated by electrophoresis and then transferred to nitrocellulose membranes. The membranes were blocked by phosphate buffer saline (PBS) containing 5% (w/v) dry skimmed milk powder and 1% Sodium Azide for 1 hour at room temperature. Then the membranes were incubated by primary antibody (rabbit polyclonal anti-podocin antibody, P0372, SIGMA, USA) overnight at 4°C. The membranes were incubated with fluorescence secondary antibodies (1 : 10,000; Jackon ImmunoResearch, anti-rabbit, 711-035-152) for 1 hour after washing the membranes with PBS which contains 0.2% Tween-20 for 5 min × 3 times. The membranes were scanned by ODYSSEY INFRARED IMAGER (Li-COR Biosciences, Lincoln, NE, USA). The scanned signal was quantitatively analyzed with Image J software (W. S. Rasband, Image J, NIH, Bethesda, MD, USA) and normalized to a loading control GAPDH (1 : 1000; Immunochemical, USA).

### 2.8. Statistical Analysis

All data are presented as the mean ± standard deviation and examined by one-way ANOVA using the GraphPad Prism software statistical package (GraphPad Software, San Diego, CA, USA). Post hoc comparisons were performed using the Student-Newman-Keuls multiple comparison test. A *P* value of less than 0.05 was considered statistically significant. All calculations were performed using SPSS version 16.0 statistical software (SPSS Inc.).

## 3. Results

### 3.1. Degradation of Cardiotoxin in the Stomach and Artificial Gastric Juice

**Figure 1:**
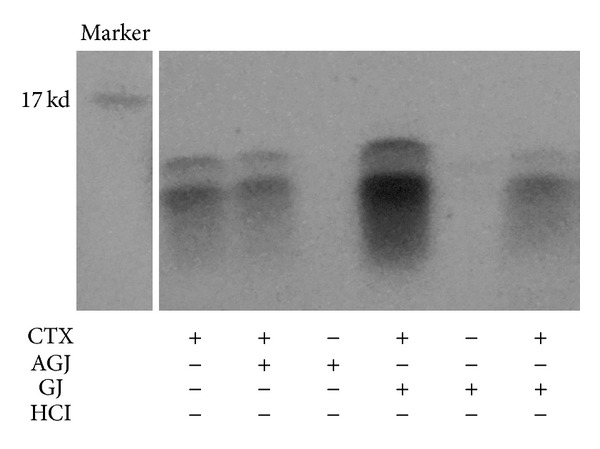
Degradation of cardiotoxin in the stomach and artificial gastric juice. The cardiotoxin was administrated into mouse stomach (2x concentration of cardiotoxin) or incubated with artificial gastric juice for 15 min. Then the degradation of cardiotoxin was assessed by dying with imperial protein stain. CTX: cardiotoxin; AGJ: artificial gastric juice; GJ: gastric juice.

As showed in Figure 1, intact cardiotoxin was detected after incubation with artificial gastric juice (AGJ) or diluted HCl for 15 min. The intact cardiotoxin was also detected in gastric juice 15 min after oral administration. In both in vitro and in vivo assays, no fragment of cardiotoxin was detected, suggesting that cardiotoxin was relatively stable in the stomach.

### 3.2. The Effects of Cardiotoxin on Body Weight and Kidney Coefficient

The changes of body weight and kidney coefficients are shown in Table 1 and Figure 3. Body weight was significantly decreased and kidney coefficients were markedly increased in the model group (adriamycin + saline) when compared with normal group (saline + saline). Body weight was slightly increased after administration of cardiotoxin for 6 weeks. Moreover, cardiotoxin significantly decreased the kidney coefficients at doses of 45, 90, and 180 *μ*g/kg, respectively (*P* < 0.001, *P* < 0.01, and *P* < 0.001). These results demonstrated that intragastric administration of cardiotoxin can reduce loss of body weight and significantly ameliorate kidney hypertrophy in the rat chronic kidney disease model.

### 3.3. The Effects of Cardiotoxin on Hypoalbuminemia, Hyperlipidaemia, and Serum Electrolyte Balance

Table 2 shows the levels of serum albumin, globulin, the radio of albumin to globulin, total cholesterol (TC), triglyceride (TG), phosphorus (P), and sodium (Na). Compared with the normal group, levels of serum albumin and sodium were decreased while globulin, total cholesterol, triglyceride, and phosphorus were increased in the model group. Cardiotoxin at a dose of 180 *μ*g/kg, a slight increase in serum albumin (17.43 ± 2.25 versus 18.49 ± 2.15 g/L), and a significant decrease in serum globulin (102.17 ± 27.14 versus 64.01 ± 26.58 g/L, *P* < 0.05) were detected. The results demonstrated that intragastric administration of 180 *μ*g/kg of cardiotoxin reduced the loss of serum albumin and significantly inhibited the increase in globulin. The levels of total cholesterol (TC) were significantly decreased after administration of 180 *μ*g/kg cardiotoxin for 6 weeks (15.05 ± 0.93 versus 11.22 ± 3.00 mmol/L, *P* < 0.01). There was no significant difference in triglyceride (TG) levels after treatment of 180 *μ*g/kg cardiotoxin even though there was a drop from 35.32 ± 11.27 to 21.70 ± 12.09 mmol/L. These results demonstrated that intragastric administration of cardiotoxin at a dose of 180 *μ*g/kg significantly ameliorated hyperlipidaemia. Besides, levels of phosphorus (P) were markedly decreased (2.95 ± 0.20 versus 2.57 ± 0.28 mmol/L; *P* < 0.01) and levels of sodium (Na) were significantly increased (135.76 ± 0.76 versus 139.13 ± 1.97 mmol/L; *P* < 0.001) after administration of 180 *μ*g/kg cardiotoxin for 6 weeks. These results suggested that cardiotoxin regulated the balance of serum electrolytes.

### 3.4. The Effects of Cardiotoxin on Proteinuria

**Figure 2:**
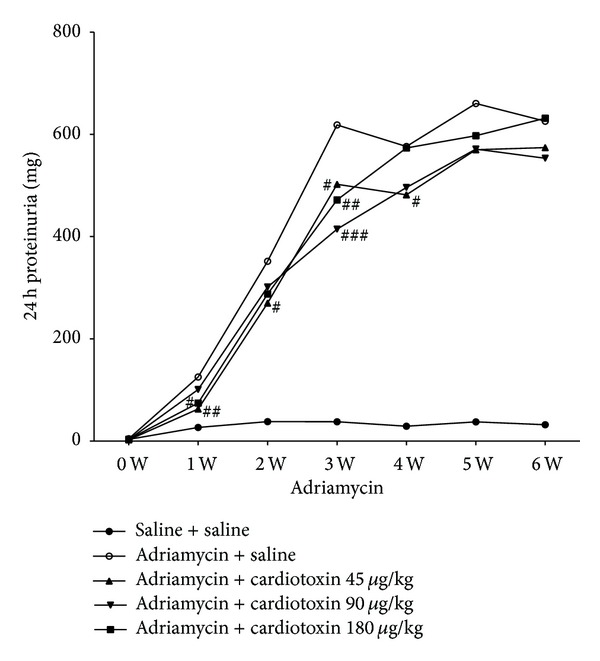
The effects of cardiotoxin on proteinuria. The rats were injected with ADR through tail vein to induce chronic kidney disease. Treatment groups were intragastrically administrated cardiotoxin at dose of 45, 90, 180 *μ*g/kg. The urine was collected for 24 hours to detect proteinuria per week. The data showing mean ± standard deviation. *P < 0.05, **P < 0.01, and ***P < 0.001 compared with “saline + saline” group. ^#^ *P* < 0.05, ^##^ *P* < 0.01, and ^###^ *P* < 0.001 compared with “adriamycin + saline” group.

**Figure 3:**
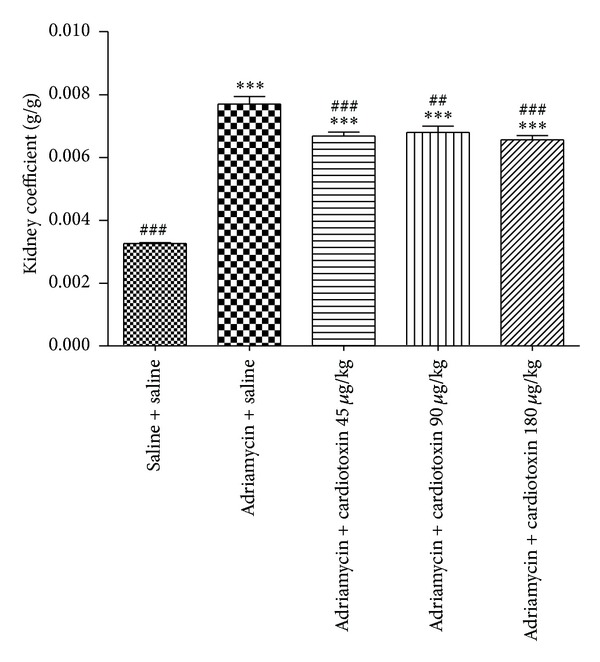
The effects of cardiotoxin on kidney coefficient. The treatment on rats was carried out as described in the legend of Figure 2. All rats were sacrificed after treatment for 6 weeks. The kidneys were removed and weighed immediately. The kidney coefficient was obtained from kidney weight divided by the body weight of rats. The data showing mean ± standard deviation. *P < 0.05, **P < 0.01, and ***P < 0.001 compared with “saline + saline” group. ^#^ *P* < 0.05, ^##^ *P* < 0.01, and ^###^ *P* < 0.001 compared with “adriamycin + saline” group.

Proteinuria is considered to be a marker of dysfunction of the glomerular filtration barrier [22]. As shown in Figure 2, urine protein was massively increased in the model group (from 125.38 ± 31.97 mg/24 hours to 625.94 ± 75.12 mg/24 hours), indicating that we successfully produced a rat model of chronic kidney disease. Although proteinuria in other groups increased as time went by, urinary protein excretion was significantly reduced after administration of cardiotoxin for 6 weeks. The protein output was 502.46 ± 123.07 (*P* < 0.05, compared with the model group), 414.76 ± 106.98 (*P* < 0.001), 471.79 ± 94.70 mg/24 hours (*P* < 0.01) after administration of cardiotoxin at doses of 45, 90, and 180 *μ*g/kg for 21 days, respectively. These results demonstrated that intragastric administration of cardiotoxin can significantly reduce proteinuria in a rat model of chronic kidney disease in the early stages.

### 3.5. The Effects of Cardiotoxin on Renal Function

**Figure 4:**
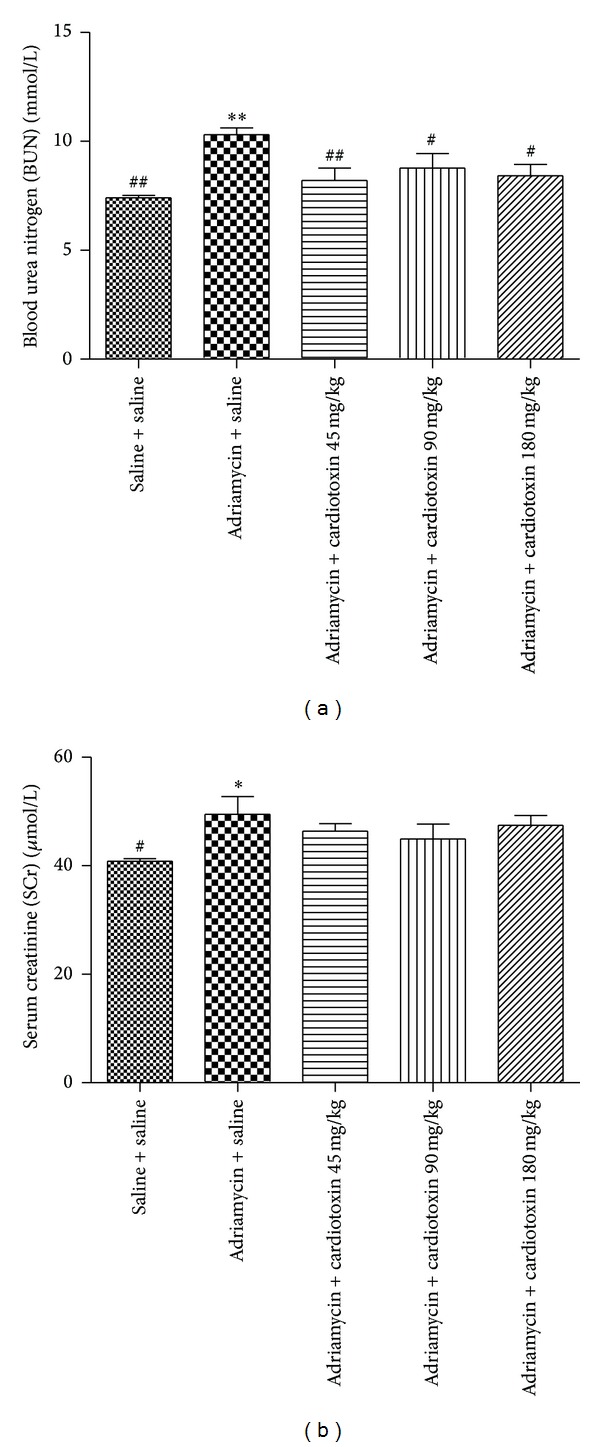
The effects of cardiotoxin on serum BUN (a) and SCr (b). The treatment on rats was carried out as described in the legend of Figure 2. All rats were sacrificed after treatment for 6 weeks. The blood was collected from abdominal aortic to detect level of BUN and SCr. The data show mean ± standard deviation. *P < 0.05, **P < 0.01, and ***P < 0.001 compared with “saline + saline” group. ^#^ *P* < 0.05, ^##^ *P* < 0.01, and ^###^ *P* < 0.001 compared with “adriamycin + saline” group.

Previous studies have shown that both BUN and SCr were increased after injection of ADR, which reflect the damage of renal function [23]. Our results showed that the levels of BUN and SCr were markedly increased in the model group (Figure 4). However, after treatment with 90 *μ*g/kg or 180 *μ*g/kg of cardiotoxin for 6 weeks, BUN was significantly decreased (10.30 ± 0.84 versus 8.42 ± 1.39 mmol/L; *P* < 0.05) and the level of SCr was slightly decreased (49.47 ± 8.69 versus 44.94 ± 7.18 *μ*mol/L). These results demonstrated that intragastric administration of cardiotoxin significantly ameliorated the renal function disorder in rat model of chronic kidney disease.

### 3.6. The Effects of Cardiotoxin on Renal Pathology

**Figure 5:**
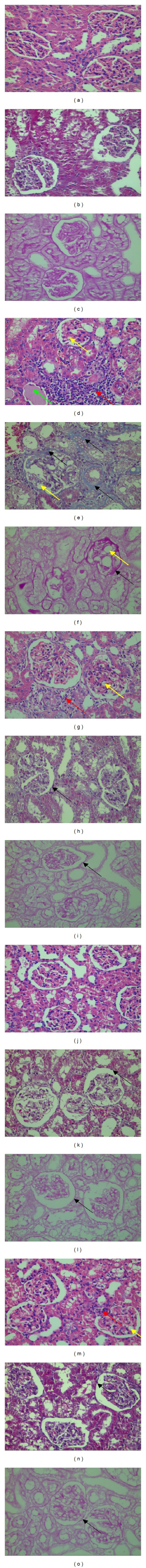
The effects of cardiotoxin on renal pathology. The treatment on rats was carried out as described in the legend of Figure 2. All rats were sacrificed after treatment for 6 weeks. The kidney was removed to make paraffin slices. (a, d, g, j, and m) showed that kidney slices stained with hematoxylin and eosin (HE). (b, e, h, k, and n) showed that kidney slices stained with Masson's trichrome. (c, f, i, l, and o) showed that kidney slices stained with periodic acid-Schif (PAS). (a, b, and c) showed the change of renal pathology in normal control group; (d, e, and f) showed the change of renal pathology in adriamycin nephropathy group. Renal pathology in treatment groups with cardiotoxin at the dose of 45 *μ*g/kg (g, h, and i), 90 *μ*g/kg (j, k, and l), and 180 *μ*g/kg (m, n, and o) can been seen, respectively. Yellow arrow showed fatty degeneration, red arrow showed inflammatory cell infiltration, green arrow showed leakage protein in tubular lumen, and black arrow showed glomerular basement membrane (GBM) thickening and tubular interstitial fibrosis.

To detect the morphological changes in ADR-induced chronic kidney disease with or without treatment with cardiotoxin, paraffin slices of kidney tissues were stained with hematoxylin and eosin (HE) and analyzed with light microscopy (Figures 5(a), 5(d), 5(g), 5(j), and 5(m)). The decrease in the number of glomeruli, occurrence of fatty degeneration (yellow arrow), tubular necrosis, inflammatory cell infiltration (red arrow), and abundant leaked protein in the tubular lumen (green arrow) were detected in the model group (Figure 5(d)). Those changes were mitigated after administration of cardiotoxin at doses of 45, 90, and 180 *μ*g/kg for 6 weeks (Figures 5(g), 5(j), and 5(m)). To further evaluate the fibrosis of tubulointerstitial tissue in ADR-induced chronic kidney disease with or without treatment with cardiotoxin, the paraffin slices were stained with Masson's trichrome (Figures 5(b), 5(e), 5(h), 5(k), and 5(n)) and periodic acid-Schiff (PAS) (Figures 5(c), 5(f), 5(i), 5(l), and 5(o)), respectively. The results showed that the model group was characterized by thick glomerular basement membrane (GBM) and tubulointerstitial collagen proliferation (black arrow) (Figures 5(e) and 5(f)). Those changes were significantly improved in the cardiotoxin administered group with Masson's trichrome staining (Figures 5(h), 5(k), and 5(n)) and periodic acid-Schiff (PAS) staining (Figures 5(i), 5(l), and 5(o)). These results demonstrated that intragastric administration of cardiotoxin reduced renal damage in a rat model of chronic kidney disease.

### 3.7. The Effects of Cardiotoxin on Podocin Expression

**Figure 6:**
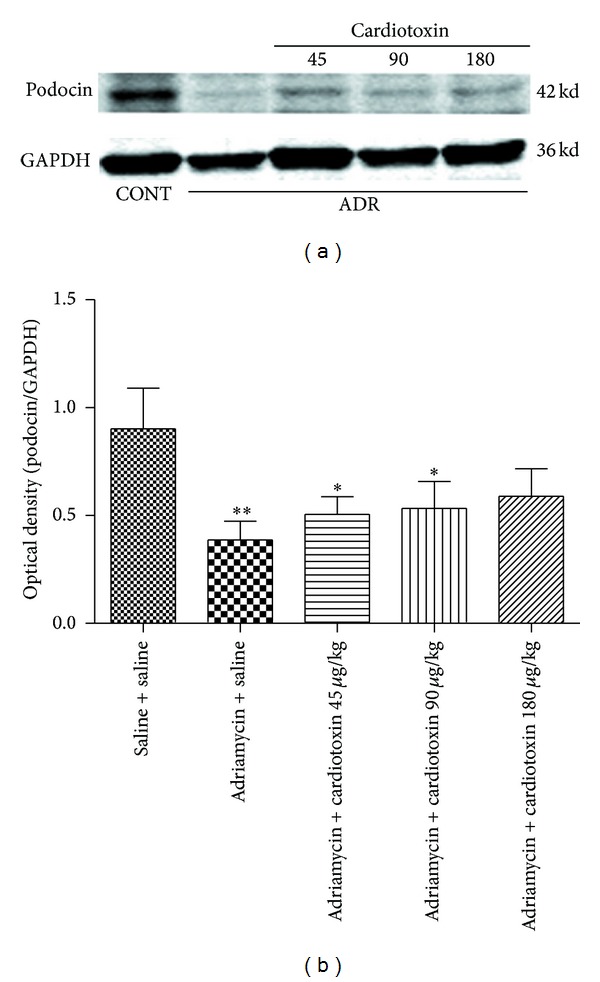
The effects of cardiotoxin on levels of renal podocin. The treatment on rats was carried out as described in the legend of Figure 2. All rats were sacrificed after treatment for 6 weeks. The level of podocin was detected by using western blot. The podocin was quantitatively analyzed with Image J software and normalized to a loading control GAPDH. The data showing mean ± standard deviation. *P < 0.05, **P < 0.01, and ***P < 0.001 compared with “saline + saline” group. ^#^ *P* < 0.05, ^##^ *P* < 0.01, and ^###^ *P* < 0.001 compared with “adriamycin + saline” group.

**Figure 7:**
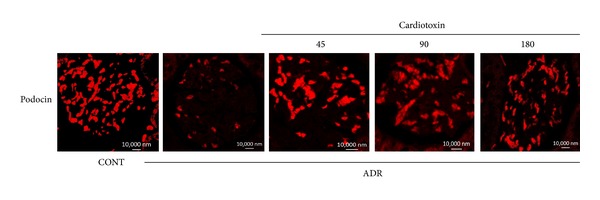
The effects of cardiotoxin on slit diaphragm protein podocin. The treatment on rats was carried out as described in the legend of Figure 2. All rats were sacrificed after treatment for 6 weeks. The kidney paraffin slices stained with antibody against podocin and analyzed by immunofluorescence. Scale bar: 10,000 nm.

Previous studies considered podocin as an important slit diaphragm protein [6, 7] and that its low expression is related to the severity of proteinuria [8]. Western blot analysis and immunofluorescence were used to detect the expression of podocin protein in the present study. The results showed that podocin protein was significantly decreased in the model group compared with the normal group. After treatment with cardiotoxin for 6 weeks, podocin protein expression was slightly increased compared with the model group, however, there was no statistical difference among the three cardiotoxin-treated groups (Figures 6 and 7).

## 4. Discussion

According to the 2010 Global Burden of Disease study, chronic kidney disease was the second fastest growing disease from 1990 to 2010. Chronic kidney disease can cause many complications, including cardiovascular disease, anemia, and mineral disorders [1]. An effective treatment of chronic kidney disease requires further development. ADR-induced nephropathy was a widely accepted rat model of chronic kidney disease, characterized by massive proteinuria, hypoalbuminemia, and hyperlibidaemia. As previously reported, we have successfully replicated a rat model of chronic kidney disease with Adriamycin [20]. We found that cardiotoxin, mainly cardiotoxin IV, significantly improved the parameters of chronic kidney disease, especially at dose of 180 *μ*g/kg. However, there was no significant difference among the three groups treated with various doses of cardiotoxin. In this study, we tested the degradation of cardiotoxin by stomach proteases after incubation of cardiotoxin with artificial gastric juice or administered to stomach. The results demonstrated that cardiotoxin was not degraded in artificial gastric juice 15 min after incubation. No obvious change of cardiotoxin was detected in gastric juice 15 min after gastric administration of cardiotoxin. No smaller fragments were detected both in vitro and in vivo assays, suggesting that the pharmacological actions of cardiotoxin were most likely produced by intact protein.

The impairment of the glomerular filtration barrier plays a critical role in the pathogenesis of chronic kidney disease. The glomerular filtration barrier consists of fenestrated endothelium, glomerular basement membrane (GBM), and slit diaphragm (SD). The integrity of the glomerular filtration barrier correlates with SD-associated protein, such as podocin. Previous research has proven that reduced expression of podocin is associated with proteinuria [8]. Thus, the therapy target on restoration of podocin levels may become a potential pathway to cure chronic kidney disease.

Proteinuria is considered to be a marker of dysfunction of the glomerular filtration barrier [22]. Our study found that intragastric administration of cardiotoxin reduced proteinuria in ADR-treated rats. Mean proteinuria was significantly reduced when compared with the ADR model group after intragastric administration of cardiotoxin for 21 days. This significant effect on reduced proteinuria was slightly weakened in the next 21 days. Histology and immunofluorescence confirmed the changes of kidney. We found abundant loss of glomerular number, severe glomerular structural damage, and low expression of podocin in ADR-induced rats. We found a less loss of glomerular number, increased expression of podocin, and improvement of glomerular structure after treatment with cardiotoxin. Thus, we speculated that cardiotoxin might have a therapeutic effect on reducing proteinuria through preventing the loss of podocin to maintain the integrity of the glomerular filtration barrier. In addition, studies have shown that body weight was decreased and kidney coefficient was increased in ADR-induced rats [23]. Our study found that cardiotoxin can increase body weight and significantly decrease kidney coefficient. Furthermore, BUN and SCr were considered to be the main index reflecting renal function and were increased after injecting ADR. Our results demonstrated that intragastric administration of cardiotoxin reduced the levels of BUN and SCr in ADR-induced rats. Therefore, cardiotoxin might have renal protective effect through amelioration of kidney hypertrophy and delaying the progression of chronic kidney disease.

Hypoalbuminemia may correlate with inflammation, malnutrition, and cardiovascular disease [24]. Research has confirmed that hypoalbuminemia was accompanied with high levels of serum globulin in ADR-induced rats [18]. The rats injected with ADR were characterized by hypoalbuminemia [25–28]. Cardiotoxin reduced the leakage of albumin and decreased serum globulin. We speculated that cardiotoxin might suppress the inflammatory and immune response, although the specific mechanism is unclear. Hyperlipidemia was considered to correlate with proteinuria and glomerulosclerosis and may accelerate the progression of kidney disease [29–31]. Our study showed that cardiotoxin decreased total triglyceride (TG) levels, especially total cholesterol (TC). Therefore, cardiotoxin may be a potential drug to treat hyperlipidemia. The rats in pathologic conditions may lose the ability to maintain electrolyte balance [32]. Previous studies have concluded that the concentration of serum phosphorus (P) was increased in ADR-induced chronic kidney disease in rats [33]. The level of phosphorus (P) was markedly decreased following cardiotoxin treatment. Meanwhile, cardiotoxin also regulated the balance of serum sodium. Thus, cardiotoxin might have a renal protective effect through the amelioration of hypoalbuminemia, hyperlipidemia, and serum electrolyte imbalance.

However, our study has inevitable limitations. Electron microscopic examination on kidney biopsies to observe the change of podocytes was absent. The dose-effect relationship of cardiotoxin was unstable. This phenomenon is common in Traditional Chinese Medicine. Furthermore, the effects of cardiotoxin on podocyte and gene expression of podocyte-associated protein require further investigation. Another issue needs to be addressed is the possibility of antibody production after long-term administration of cardiotoxin of causing allergy and loss of efficacy of cardiotoxin, even though the current information suggests that the possibility is low.

## 5. Conclusion

In the present study, we tentatively concluded that intragastric administration of cardiotoxin can ameliorate symptoms of ADR-induced chronic kidney disease in rats, including proteinuria,hypoalbuminemia, and hyperlipidaemia. Cardiotoxin may reduce proteinuria, though lowering the loss of slit diaphragm protein podocin to maintain the integrity of the glomerular filtration barrier. These findings suggest that cardiotoxin is an active ingredient of NNAV in protecting adriamycin nephropathy. Cardiotoxin itself or in combination with other active components of NNAV may be used for treatment of chronic kidney disease.

## Figures and Tables

**Table 1:** The effect of cardiotoxin on body weight.

Items	Saline + saline	Adriamycin + saline	Adriamycin + cardiotoxin 45 *μ*g/kg	Adriamycin + cardiotoxin 90 *μ*g/kg	Adriamycin + cardiotoxin 180 *μ*g/kg
First week	183.80 ± 4.55	166.29 ± 6.73***	155.00 ± 5.54^∗∗∗##^	157.29 ± 4.54^∗∗∗##^	158.14 ± 7.36^∗∗∗#^
Second week	213.80 ± 8.61	178.14 ± 5.73***	169.57 ± 7.23***	171.86 ± 4.67***	173.00 ± 13.49***
Third week	271.40 ± 10.24	203.71 ± 8.96***	204.14 ± 10.43***	197.57 ± 9.25***	206.57 ± 13.15***
Fourth week	319.00 ± 12.59	220.43 ± 10.39***	225.00 ± 13.80***	222.71 ± 8.96***	224.43 ± 11.33***
Fifth week	337.00 ± 13.34	214.71 ± 7.34***	226.43 ± 12.73***	218.43 ± 6.65***	226.14 ± 14.00***
Sixth week	368.40 ± 14.60	223.00 ± 11.30***	234.71 ± 16.93***	216.71 ± 23.51***	234.57 ± 15.23***

Note. The data showing mean ± standard deviation. ***P < 0.001 compared with “saline + saline” group. ^#^ *P* < 0.05, ^##^ *P* < 0.01, compared with “adriamycin + saline” group.

**Table 2:** The effects of cardiotoxin on serum bioparameters.

Items	Saline + saline	Adriamycin + saline	Adriamycin + cardiotoxin 45 *μ*g/kg	Adriamycin + cardiotoxin 90 *μ*g/kg	Adriamycin + cardiotoxin 180 *μ*g/kg
Albumin (g/L)	30.06 ± 0.89^###^	17.43 ± 2.25***	17.74 ± 1.78***	16.90 ± 2.89***	18.49 ± 2.15***
Globulin (g/L)	23.60 ± 0.79^###^	102.17 ± 27.14***	83.07 ± 30.88**	92.01 ± 43.87***	64.01 ± 26.58^∗#^
Albumin/Globulin	1.28 ± 0.07^###^	0.19 ± 0.09***	0.25 ± 0.14***	0.26 ± 0.20***	0.35 ± 0.18***
Triglyceride (TG) (mmol/L)	1.21 ± 0.12^###^	35.32 ± 11.27***	27.90 ± 12.70**	31.20 ± 17.41***	21.70 ± 12.09**
Total cholesterol (TC) (mmol/L)	1.61 ± 0.12^###^	15.05 ± 0.93***	11.95 ± 2.38^∗∗∗#^	12.88 ± 2.69***	11.22 ± 3.00^∗∗∗##^
Phosphorus (P) (mmol/L)	2.77 ± 0.29	2.95 ± 0.20	2.86 ± 0.15	2.69 ± 0.17^#^	2.57 ± 0.28^##^
Sodium (Na) (mmol/L)	138.92 ± 0.50^##^	135.76 ± 0.76**	137.90 ± 1.44^#^	138.97 ± 2.13^##^	139.13 ± 1.97^###^

Note: the data showing mean ± standard deviation. *P < 0.05, **P < 0.01, and ***P < 0.001 compared with “saline + saline” group. ^#^ *P* < 0.05, ^##^ *P* < 0.01, and ^###^ *P* < 0.001 compared with “adriamycin + saline” group.
